# Form of Supplemental Selenium in Vitamin-Mineral Premixes Differentially Affects Early Luteal and Gestational Concentrations of Progesterone, and Postpartum Concentrations of Prolactin in Beef Cows

**DOI:** 10.3390/ani10060967

**Published:** 2020-06-03

**Authors:** Sarah Carr, Yang Jia, Benjamin Crites, Charles Hamilton, Walter Burris, J. Lannett Edwards, James Matthews, Phillip J. Bridges

**Affiliations:** 1 Department of Animal and Food Sciences, University of Kentucky, Lexington, KY 40546, USA; sarah.carr@uky.edu (S.C.); yang.jia@uky.edu (Y.J.); benjamin.crites@uky.edu (B.C.); chamilto@uky.edu (C.H.); roy.burris@uky.edu (W.B.); james.matthews@uky.edu (J.M.); 2 Department of Animal Science, University of Tennessee, Institute of Agriculture, AgResearch, Knoxville, TN 37996, USA; jedwards@utk.edu

**Keywords:** corpus luteum, progesterone, prolactin, gestation, lactation, selenium

## Abstract

**Simple Summary:**

Soils with inadequate levels of selenium are widespread in the northwest, northeast, and southeast USA. Therefore, dietary supplementation of forage-grazing beef cattle with additional selenium is recommended in these regions for optimal growth, immune function, and fertility. We have reported that the form of selenium provided to Angus-cross cows can affect circulating concentrations of progesterone on day 6 of the estrous cycle, a time when increased progesterone is known to promote fertility. Hence, we sought to confirm this initial finding, determine the effect of the form of selenium on peripheral concentrations of progesterone during gestation, and determine the effect of the form of selenium on circulating concentrations of prolactin during lactation. Cows were supplemented with equimolar amounts of either an inorganic form, or a 1:1 mixture of inorganic and organic forms of selenium throughout this study. We confirmed our original finding that the mixed (1:1 inorganic to organic selenium) supplement increased systemic progesterone in the early luteal phase of the estrous cycle, and determined that cows maintained on this same supplement had elevated concentrations of progesterone throughout gestation. Interestingly, these same cows revealed a treatment-induced decrease in systemic prolactin during late lactation. The form of selenium provided to cows can be manipulated to affect reproductive responses and offers a viable management tool to improve fertility in cows in regions with selenium-deficient soils.

**Abstract:**

Soils with marginal to deficient levels of selenium (Se) are widespread in the northwest, northeast, and southeast US. Supplementation to the diet of forage-grazing beef cattle with a vitamin-mineral mix containing additional Se is recommended in these geographic regions. We have reported that the form of supplemental Se provided to Angus-cross beef cows can affect circulating levels of progesterone (P4) on day 6 of the estrous cycle, a time when increased P4 is known to promote fertility. The objectives of this study were to (1) confirm and expand upon our initial report that the form of Se provided to cows affects early luteal-phase concentrations of systemic P4, (2) determine the effects of the form of Se on concentrations of P4 during gestation, and (3) determine the effects of the form of Se on concentrations of prolactin (PRL) during lactation. Throughout this study, Angus-cross beef cows had ad libitum access to a vitamin-mineral mix containing 35 ppm of Se in either an inorganic form (ISe) or a 1:1 mix of inorganic and organic forms (MIX). We observed a MIX-induced increase (*p* = 0.006) in systemic concentrations of P4 on day 7 but not on days 4 or 10 of the estrous cycle, consistent with our earlier report. We observed a MIX-induced increase (*p* = 0.02) in the systemic concentration of P4 at months 1, 3, 5, and 7 of gestation, and a MIX-induced decrease (*p* < 0.05) in systemic concentrations of PRL at months 5 and 6 of lactation. In summary, the form of Se provided to cows can be manipulated to affect the early luteal phase and gestational concentrations of P4, and postpartum concentrations of PRL.

## 1. Introduction

Dietary selenium (Se) is required for the synthesis of selenoproteins, including glutathione peroxidases and thioredoxin reductases, which catalyze the breakdown of hydrogen peroxide and lipid hydroperoxides, protecting extracellular and intracellular cell membranes [1,2,3,4]. In cattle, a deficiency in Se has been shown to reduce rates of growth [5], alter immune responses [6,7], and reduce indicators of fertility [8], including an increased rate of abortion and perinatal mortality [9]. Soils with marginal to deficient levels of Se are widespread in the northwest, northeast, and southeast USA [10]. Because the content of Se in forages is dependent upon the content of Se in the soil, supplementation to the diet of forage-grazing beef cattle with a vitamin-mineral mix containing Se is recommended in these areas.

Conventionally, free-choice vitamin-mineral mixes containing Se have been formulated with an inorganic form of Se, sodium selenate or sodium selenite. However, the available forms of Se that naturally occur in forages are the organic forms, selenomethionine and selenocysteine [11]. The form of Se available to an animal affects the bioavailability of Se in blood and tissues, and the bioactivity of Se by affecting blood glutathione peroxidases [12,13,14]. Additionally, organic forms of supplemental Se have been shown to stimulate tissue assimilation as noted by the upregulation of mRNA associated with genes promoting cellular growth, proliferation, and development [12,13].

We previously demonstrated that the consumption of equimolar amounts of Se in organic (OSe), inorganic (ISe), or mixed (MIX, 1:1 OSe:ISe) forms by beef cows throughout pregnancy resulted in distinct transcriptome profiles in the testes collected from their newborn bull calves, including the differential expression of mRNAs known to regulate gonadal steroidogenesis [15], and that cows consuming MIX versus ISe had an increased concentration of systemic progesterone (P4) on day 6 of the estrous cycle [16]. Elevated early luteal phase concentrations of P4 have been reported to advance endometrial development [17], increase embryonic length [18,19], and improve rates of pregnancy [20], suggesting that the form of Se supplied to cattle may be used to manipulate early luteal phase concentrations of P4 in a manner that promotes fertility.

Given these documented effects of the form of Se on steroidogenic gene expression in the neonatal testes, and on day 6 concentrations of P4 in the cycling cow, the first objectives of this study were to (1) confirm and expand upon the timing of our initial report of an MIX-induced increase in early luteal phase P4, and (2) to quantify the effects of the form of Se on concentrations of P4 throughout gestation itself. We also reported that the form of Se supplemented to steers affects the systemic concentration of prolactin (PRL) [21]. Although PRL is most widely recognized for its role in the development of the mammary gland and the induction of lactation [22], it has been further identified as a regulator of multiple physiological processes, including growth and development, metabolism, and immune function [23,24]. Therefore, our third objective was to determine the effects of the form of Se on concentrations of PRL during lactation. Collectively, we hypothesized that the MIX form of Se, versus ISe, would increase concentrations of P4 throughout gestation, and then PRL during lactation. From this research, supplementation strategies using a defined form of Se may be adopted by beef producers to influence endocrine pathways that promote the establishment and maintenance of pregnancy in cows, followed by the growth and development of their offspring prior to weaning.

## 2. Materials and Methods 

### 2.1. Animals and Experimental Procedure

All animal research protocols were approved by the University of Kentucky Institutional Animal Care and Use Committee (IACUC #2017-2828). Multiparous Angus-cross cows (4–11 years of age) were managed in a forage-based fall-calving production system, and housed at the University of Kentucky Research and Education Center in Princeton, Kentucky. With the exception of months 5 to 7 of lactation, when all cows consumed a common silage ration, all animals continuously grazed endophyte-infected tall fescue pastures. 

For the experiments described herein, cows were randomly selected from pre-existing Se form-specific cow herds. As described before [15,16,25,26], each cow had ad libitum access to a free-choice vitamin-mineral mix formulated to contain 35-ppm ISe (Sodium selenite; Prince Agri Products, Inc., Quincy, IL, USA) or a 1:1 combination (MIX) of ISe and OSe (SEL-PLEX; Alltech, Inc., Nicholasville, KY, USA) for the duration of this study. Details of individual ad libitum intake and the effects on blood Se have been previously reported [24], as has the composition of the basal vitamin-mineral mix [21].

### 2.2. Experimental Regimen

#### 2.2.1. Effect of Form of Se on Early Luteal Phase Concentrations of P4

To expand upon our earlier report that the MIX form of Se increased the concentration of systemic P4 on day 6 post-estrus, we determined the effect of supplementation with MIX versus ISe on concentrations of P4 on day 4, 7, and 10 post-estrus, spanning the interval where increased P4 is known to promote fertility [18,27,28,29,30]. Briefly, luteal function was confirmed in 24 cows (*n* = 12 per treatment) by transrectal ultrasonography using a 5–8 MHz, 66-mm linear array transducer (Ibex Pro, E.I. Medical Imaging, Loveland, CO, USA). Cows were then administered i.m. with 25 mg prostaglandin F2α (PGF2α; Lutalyse, Pfizer Animal Health, New York, NY, USA) to induce regression of the corpus luteum (CL) and monitored for behavioral estrus (day 0). On days 4, 7, and 10 post-estrus, 8 mL of blood was collected via jugular venipuncture into sodium-heparin-containing tubes (Vacutainer, Becton, Dickinson and Company, Franklin Lakes, NJ, USA) for retrieval and quantification of plasma concentrations of P4 by radioimmunoassay [31].

#### 2.2.2. Effect of Form of Se on Concentrations of P4 during Gestation

To determine the effect of supplementation with MIX versus ISe on the concentration of systemic P4 during gestation, estrous was synchronized in cows using an intravaginal Controlled Internal Drug Releasing (CIDR) device (ZOETIS EAZI-BREED™ CIDR ® 1.38 g progesterone, Zoetis, Parsippany, NJ, USA) for 7 days, with each cow administered 25 mg of PGF2α at CIDR removal. At observed estrus, cows were artificially inseminated by an experienced technician. Pregnancy was confirmed via transrectal ultrasonography at 45 days after insemination, and only cows that conceived to artificial insemination (AI) were included in this study (ISe, *n* = 12; MIX, *n* = 14). At months 0, 1, 3, 5, and 7 of gestation, 8 mL of blood was collected via jugular venipuncture into sodium-heparin-containing tubes for retrieval and quantification of plasma concentrations of P4 by radioimmunoassay (RIA) [31].

#### 2.2.3. Effect of Form of Se on Concentrations of PRL during Lactation

To determine the effect of supplementation with MIX versus ISe on the circulating concentration of PRL during the postpartum period, sampling of the same cows that were used to determine the effect of treatment on gestational concentrations of P4 was continued (*n* = 12 per treatment, as 2 cows were removed from this study due to management considerations). Beginning at 1 month postpartum, 8 mL of blood was collected via jugular venipuncture into additive-free tubes (Vacutainer, Becton, Dickinson and Company, Franklin Lakes, NJ, USA) every 28 days for the following 6 months for the subsequent retrieval and quantification of serum concentrations of PRL by RIA [32].

### 2.3. Se and Hormone Analyses

To verify the Se-adequate status of cows during this trial, whole blood was retrieved from cows throughout this study for subsequent analysis of total blood Se. We previously reported that liver and plasma levels of Se stabilize between 54 and 112 days of supplementation [13]. Total blood Se was determined by Michigan State University Diagnostic Center for Population and Animal Health (DCPAH) using an Agilent 7900 inductively coupled plasma-mass spectrometer, as described previously [33]. Concentrations of P4 were quantified in samples of plasma by a commercially available competitive RIA without extraction (ImmuChem™ Coated Tube Progesterone 125-I RIA Kit, MP Biomedicals, Costa Mesa, CA, USA), as described previously [31]. Low (0.2 ng/mL), medium (1.6 ng/mL), and high (4 ng/mL) reference samples were included in the RIA. All samples were analyzed within a single assay, and the intra-assay coefficient of variation (CV) was 3.15%. 

Concentrations of PRL were quantified by the laboratory of Dr. Lannett Edwards (University of Tennessee), using a double-antibody RIA as described previously [34]. Low (5 ng/mL) and high (10 ng/mL) reference samples were included in the RIA. The intra-assay CV was 4.49% and the inter-assay CV was 8.59%.

### 2.4. Statistical Analysis

The individual cow was the experimental unit. To determine the effect of the form of Se on concentrations of circulating P4 and PRL, data were subjected to ANOVA with repeated measures using the PROC Glimmix function of SAS statistical software package (version 9.4; SAS Institute, Inc. Cary, NC, USA). Results are presented as the least square means ± standard error of the mean (LS Means ± SEM). Significance was declared at *p* < 0.05, and a tendency to differ was declared when 0.05 < *p* < 0.10.

## 3. Results

### 3.1. Concentrations of Se in Whole Blood

All cows were maintained on the form of Se-specific treatments that provided adequate concentrations of whole blood Se (0.14 to 0.17 ± 0.01 ng/mL) throughout the duration of this study [35,36].

### 3.2. Concentrations of P4 during the Early Luteal Phase

The concentrations of P4 were determined in plasma collected from cows on days 4, 7, and 10 of the estrous cycle. Cows maintained on the MIX treatment group versus ISe had a greater concentration of systemic P4 on day 7 (*p* = 0.006) but not on days 4 or 10 (*p* > 0.05) post-estrus (Table 1). 

### 3.3. Concentrations of P4 during Gestation 

**Figure 1:**
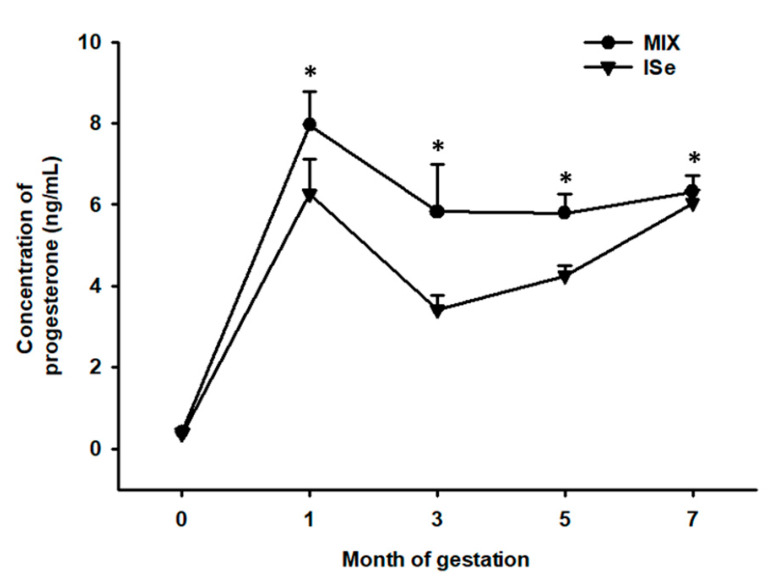
Effect of the form of Se on the systemic concentration of P4 during gestation. Cows had ad libitum access to vitamin-mineral premixes containing 35 ppm of either inorganic (ISe; sodium selenite; *n* = 12), or a 1:1 combination (MIX) of ISe and OSe (SEL-PLEX; *n* = 14) selenium. The concentrations of P4 was affected by treatment (*p* = 0.02) and time (*p* < 0.001) but not treatment by time (*p* = 0.2). * The concentration of P4 was elevated in MIX-supplemented cows at months 1, 3, 5 and 7 of gestation (*p* < 0.05).

The concentrations of P4 were determined in plasma collected from cows at months 0, 1, 3, 5, and 7 of confirmed pregnancy. Cows maintained on the MIX treatment group versus ISe had a greater (*p* = 0.02) concentration of systemic P4 at months 1, 3, 5, and 7 of gestation (Figure 1).

### 3.4. Concentrations of PRL during Lactation 

**Figure 2:**
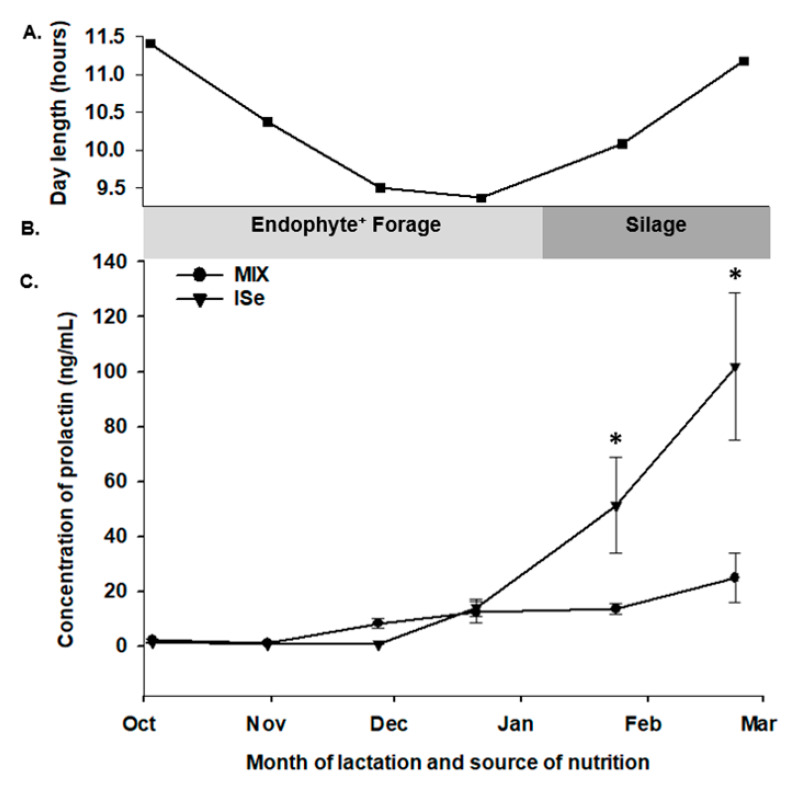
Effect of the form of Se on the systemic concentration of prolactin (PRL) during lactation. (**A**) Day length (hours), (**B**) basal diet (endophyte-infected tall fescue pasture versus silage), and (**C**) serum concentrations of PRL during lactation. Cows had ad libitum access to vitamin-mineral premixes containing 35 ppm of either inorganic (ISe; sodium selenite; *n* = 12), or a 1:1 combination (MIX) of ISe and OSe (SEL-PLEX; *n* = 12) selenium. The concentration of PRL was affected by time (*p* < 0.001) and treatment by time (*p* < 0.001) but not by treatment (*p* = 0.8). * The concentration of PRL was elevated in ISe-supplemented cows in the 5th and 6th periods (February and March).

Beginning at 1 month postpartum, the concentration of PRL was determined in serum retrieved from cows every 28 days for the following 6 months. Prolactin was affected by time (*p* < 0.001) and treatment by time (*p* < 0.001) and tended to be affected by treatment (*p* = 0.08). The form of Se did not affect (*p* > 0.05) the concentration of PRL in the systemic blood of cows during the first four (28 day) periods after calving. However, cows maintained on the MIX treatment group versus ISe had a lower (*p* < 0.05) concentration of systemic PRL during the fifth and sixth periods (February and March, Figure 2).

## 4. Discussion

The objectives of this study were to determine, in Se-adequate cattle, the effect of the form of supplemental Se on concentrations of P4 during the early luteal phase of the estrous cycle and gestation, then on concentrations of PRL during lactation. Analysis of the concentrations of Se in the whole blood of cows during this study confirmed that these cows were maintained in an Se-adequate status throughout this study [35,36].

We previously reported that supplementing cows with the mixed blend of organic and inorganic Se (MIX), versus inorganic Se (ISe) alone, resulted in an increase in peripheral concentrations of P4 by 50% on day 6 of the estrous cycle [16]. Herein, we confirm this stimulatory effect of the form of Se on early luteal phase P4, with MIX now reported to increase P4 on days 6 [16] and 7 (herein) but not days 4 or 10 of the estrous cycle. Multiple reports have indicated that increases in early luteal phase concentrations of P4 advance endometrial development, increase embryonic length, and improve rates of pregnancy. For example, Beltman et al. [37] artificially increased P4 in heifers from day 3.5 to 6 or day 4.5 to 8 post-estrus. In both instances, they reported a significant relationship between the change in serum P4 and embryo survival rate. Consistent with this, Mann et al. [38] determined that P4 on days 4 and 5 differed among cows that had a large well-elongated embryo versus a less well-elongated embryo. Carter et al. [29] artificially increased P4 in heifers from days 3 to 7 post-estrus and reported P4-dependent changes to the transcriptome of day 7-recovered blastocysts, and Forde et al. [39] increased P4 in heifers from day 3 post-estrus and reported P4-dependent divergence of the endometrial transcriptome by day 7, as well as a P4-dependent increase in conceptus development by day 14. Importantly, Yan et al. [40] performed a comprehensive meta-analysis from the data reported from 53 publications and concluded that increased P4 (treatment with P4 initiated between days 3 and 7) post-estrus significantly increased the chance of pregnancy. With the MIX form of Se now shown to increase day 6 and 7 concentrations of systemic P4, we therefore confirm a novel producer-friendly management technique to increase the levels of circulating P4 at a time that is known to promote endometrial function, embryonic development, and conceptus survival.

In the current study, concentrations of P4 remained higher throughout gestation in the MIX- versus ISe-supplemented cows (Figure 1). For over a century, the requirement of P4 for the maintenance of pregnancy has been understood [41], with removal of the CL (the primary source of P4 in the cow) prior to 200 days of gestation resulting in the termination of pregnancy [42,43,44,45]. However, the physiological importance of the MIX-induced increase in gestational levels of P4 observed herein is still hard to define. Although the level of gestational P4 has been reported to affect the incidence of retained placenta in dairy cows [46,47,48], with Erb et al. [49] concluding that high levels of P4 in the blood are necessary for normal gestation and expulsion of fetal membranes, whether the MIX-induced increase in gestational levels of P4 observed herein provides a positive benefit to the maintenance of pregnancy in beef cows remains to be determined.

It is well established that the concentration of circulating PRL is affected by the photoperiod and temperature [50,51], as well as exposure to endophyte-infected tall fescue forages [52,53,54]. Worthy to note, a decrease in circulating concentrations of PRL is considered a hallmark of endophyte-induced fescue toxicosis [53]. Interestingly, the divergence in concentrations of PRL that we observed in cows on the MIX versus ISe treatment groups occurred during the winter months when all cattle were maintained on a common corn-based silage ration in lieu of grazing endophyte-infected tall fescue pastures. While the mechanism responsible for the more robust increase in PRL in Ise- versus MIX-supplemented cows in this study is unclear, we reported that in steers grazing endophyte-infected tall fescue pastures during the summer months and supplemented with either ISe or MIX, MIX-supplemented steers had increased concentrations of serum PRL [21]. Whether the contrasting results in PRL between these two trials is reflective of differences in gender (steers versus cows) or diet (pasture versus silage) remains to be determined. 

Although PRL is most recognized for its role in the development of the mammary gland and the induction of lactation [22], more recently defined are the pleiotrophic roles of PRL, including effects on immune function and osmoregulation [23,24]. Extra-pituitary sources of PRL have been identified and include immune cells (macrophages, B cells, NK cells, T cells, thymocytes, and peripheral blood mononuclear cells [55,56,57,58]), with the PRL receptor also localized widely throughout the immune system [59]. Prolactin also affects water transport across amniotic membranes, and PRL-induced solute transport during late pregnancy may be an important preparatory player for subsequent lactation [60]. We reported that the form of Se affects the expression of >500 annotated genes in the pituitary gland of beef steers [54], with a functional analysis of that microarray-based dataset revealing that the form of Se predominately affected a canonical pathway network between PRL and pro-opiomelanocortin (POMC), adrenocorticotropic hormone (ACTH), and α-melanocyte-stimulating hormone (α-MSH) synthesis-related hormones. Overall, it appears that there is an interplay between a direct effect of the form of Se on the bovine pituitary gland and its release of PRL, and the form of Se-mediated PRL-immune cell signaling. 

## 5. Conclusions

In this report, we aimed to (1) confirm our initial report of an MIX-induced increase in early luteal phase concentrations of systemic P4, (2) define the effects of the form of Se on P4 throughout gestation, and (3) quantify the effects of the form of Se on concentrations of PRL during lactation. Even with the limitation of animal numbers, our hypothesis that the MIX treatment would increase circulating concentrations of P4 during gestation and PRL during lactation was confirmed for P4 but not for PRL in which the reverse was observed. With increased early luteal phase P4 known to promote fertility, and the absolute requirement for P4 on the maintenance of a pregnancy, it can be concluded that supplementation with the MIX form of Se can be considered a viable management tool to improve fertility in cows maintained in regions where the Se content of soils is inadequate and supplementation with this trace mineral is recommended. Whether manipulation of the form of Se to affect postpartum concentrations of PRL can be used as a management tool to promote the growth, development, and immune function of suckling calves remains to be fully elucidated.

## Figures and Tables

**Table 1:** Effect of the form of supplemental Se on the concentration of progesterone (P4) in the peripheral plasma of cows during the early luteal phase of the estrous cycle ^1.^

Variable	Treatment		*p*-Value ^2^
	ISe	MIX	
	Mean ± SEM	Mean ± SEM	
Progesterone (ng/mL)			
Cerny et al., 2016 *			
No. of cows (n)	9	9	
Day 6 ^†^	3.44 ± 0.18 ^a^	5.14 ± 0.60 ^b^	0.035
*Experimental Study*			
No. of cows (n)	12	12	
Day 4	1.02 ± 0.22	0.94 ± 0.12	0.740
Day 7 ^†^	2.92 ± 0.27 ^a^	3.91 ± 0.16 ^b^	0.006
Day 10	7.17 ± 0.54	6.36 ± 0.55	0.308

^1^ Selenium was supplemented at 35 ppm as either inorganic (ISe; sodium selenite), or a 1:1 combination (MIX) of ISe and organic (OSe, SEL-PLEX). Selenium was provided ad libitum in free-choice vitamin-mineral premixes; ^2^ *p*-Values associated with one-way ANOVA; * Adapted from results reported in Cerny et al., 2016; **^†^** Means ± SEM with different superscripted letters (a,b) differ (*p* ≤ 0.05).
